# Home Environmental Factors Associated with Health Outcomes in Community-Dwelling Older Adults

**DOI:** 10.1093/geroni/igaf122.3098

**Published:** 2025-12-31

**Authors:** Youngmin Cho, Jing Huang, Jiaying Li, Claire Wang, Russell Calderon, Michelle Liu, Katherine Ornstein, Junxin Li

**Affiliations:** Johns Hopkins University, Baltimore, Maryland, United States; Johns Hopkins University, Baltimore, Maryland, United States; Johns Hopkins University, Baltimore, Maryland, United States; Johns Hopkins University, Baltimore, Maryland, United States; Johns Hopkins School of Medicine, Baltimore, Maryland, United States; Johns Hopkins University, Baltimore, Maryland, United States; Johns Hopkins University, Baltimore, Maryland, United States; Johns Hopkins University, Baltimore, Maryland, United States

## Abstract

Older adults spend a substantial proportion of their time indoors, making them particularly vulnerable to home environmental factors. However, existing research has focused on outdoor exposures near residential addresses, leaving a critical gap in understanding the scope of indoor environmental impacts on aging. We investigate the associations between indoor environment and health outcomes (sleep, mental, and cognitive health) in community-dwelling older adults without dementia, using the baseline data from an ongoing RCT. Indoor environment includes self-reported environmental comfort—encompassing light, air quality, temperature, and noise—and continuous 24/7 sensor-based measurements of air pollution (PM_2.5_, CO_2_, NO_x_, and TVOCs), humidity, temperature, light, and sound levels. Sleep health was assessed via self-reported measures (Pittsburgh sleep quality index [PSQI], and insomnia severity) and objective measurement devices—ActiGraph and ambulatory EEG. Cognitive function was evaluated using the Everyday cognition scale (ECog) and the NIH Toolbox, while mental health was measured through the Geriatric depression scale, Geriatric anxiety scale, and Perceived stress scale. Our findings (n = 39) showed lower perceived environmental comfort was significantly associated with poorer sleep quality (PSQI, r = 0.36), increased insomnia severity (r = 0.32), worsened mental health (depression, r = 0.34; anxiety, r = 0.36; stress, r = 0.35), and lower cognitive function (ECog, r = 0.32). Additionally, increased evening light exposure correlated with higher stress (r = 0.75), higher CO_2_ levels were associated with poorer delayed recall (r=-0.64), and higher humidity was linked to increased insomnia severity (r = 0.64). These findings highlight the need for targeted interventions to improve indoor environmental conditions, which may mitigate risks to sleep, mental, and cognitive health in community-dwelling older adults.

